# The Differences in the Characteristics of Insulin-producing Cells Using Human Adipose-tissue Derived Mesenchymal Stem Cells from Subcutaneous and Visceral Tissues

**DOI:** 10.1038/s41598-019-49701-0

**Published:** 2019-09-13

**Authors:** Yuma Wada, Tetsuya Ikemoto, Yuji Morine, Satoru Imura, Yu Saito, Shinichiro Yamada, Mitsuo Shimada

**Affiliations:** Department of Surgery, Graduate School of Biomedical Sciences, Tokushima University, Tokushima, Japan

**Keywords:** Pancreatic disease, Preclinical research

## Abstract

The aim of this study was to investigate the characteristics of insulin producing cells (IPCs) differentiated from adipose-tissue derived stem cells (ADSCs) isolated from human subcutaneous and visceral adipose tissues and identify ADSCs suitable for differentiation into efficient and functional IPCs. Subcutaneous and visceral adipose tissues collected from four (4) patients who underwent digestive surgeries at The Tokushima University (000035546) were included in this study. The insulin secretion of the generated IPCs was investigated using surface markers by: fluorescence activated cell sorting (FACS) analysis; cytokine release; proliferation ability of ADSCs; *in vitro* (glucose-stimulated insulin secretion: (GSIS) test/*in vivo* (transplantation into streptozotocin-induced diabetic nude mice). The less fat-related inflammatory cytokines secretions were observed (*P* < 0.05), and the proliferation ability was higher in the subcutaneous ADSCs (*P* < 0.05). Insulin expression and GISI were higher in the subcutaneous IPCs (*P* < 0.01 and *P* < 0.05, respectively). The hyperglycaemic state of all mice that received IPCs from subcutaneous fat tissue converted into normo-glycaemia in thirty (30) days post-transplantation (4/4,100%). Transplanted IPCs were stained using anti-insulin and anti-human leukocyte antigen antibodies. The IPCs generated from the ADSCs freshly isolated from the human fat tissue had sufficient insulin secreting ability *in vitro* and *in vivo*.

## Introduction

Type 1 diabetes mellitus (T1DM) is a chronic auto-immune disorder characterized by the destruction of pancreatic β-cells due to insulitis and absolute insulin deficiency. Islet transplantation (ITx) is one of the treatment choices for freeing people with TIDM from a lifelong dependence on insulin injections and results in reducing the risk of hypoglycaemic events and other serious complications. However, it has been reported that ITx has a low insulin independent rate and there is difficulty in securing sufficient islets due to a severe donor shortage in some countries^1,2^. The possibility to resolve these urgent issues lies in the generation of insulin producing cells (IPCs) derived from mesenchymal stem cells (MSCs). Regarding the harvest location of MSCs, it has been reported that adipose-tissue derived stem cells (ADSCs) can be obtained by a less invasive procedure with lower ethical problems compared to other MSCs^3–5^. Thus, the focus of this study is on the ADSCs as a new cell source of MSCs^6–8^ and the establishment of a new 2-step protocol for the differentiation of IPCs from ADSCs with a short culture duration and functional efficiency^9^. Moreover, this 2-step protocol was modified into a xeno-antigen free and three-dimensional (3D) culture protocol^10,11^ which improved cell quality and function *in vitro*/*in vivo* and resulted in the normalization of diabetic nude mice blood glucose over 120 days^12^. In other words, this modified protocol is reaching the pre-clinical level of proof of a concept.

Accordingly, when considering the generation and clinical application of IPCs it is important to investigate optimal adipose tissue serving as the cell source. Therefore, this study currently focused on the procurement location of adipose tissue. The adipose tissue accumulates mainly in subcutaneous and visceral locations. It has already been reported that the microenvironments of adipose tissue such as immune-cells and various cytokines secreted from them differ, depending on the location of the adipose tissue^13–16^. It is also reported that subcutaneous adipose cells are smaller in size and higher in differentiation and proliferation ability compared to visceral adipose cells^13^. In addition, compared with visceral adipose tissue, there are three (3) times or more B cells in subcutaneous adipose tissue and these B cells suppress the activity of CD8^+^ T cells via IL-10 secretion and M1 macrophage which induce an inflammatory response^16^. Therefore, there must be various functional and cell-fatal differences depending on the location of the adipose tissue due to the differences in the microenvironment.

In this study IPCs generated from ADSCs isolated from fresh human subcutaneous and visceral adipose tissues were characterized and the identification which ADSCs can achieve to differentiate into more efficient and functional IPCs was shown.

## Results

### The characteristic differences of isolated ADSCs

**Figure 1:**
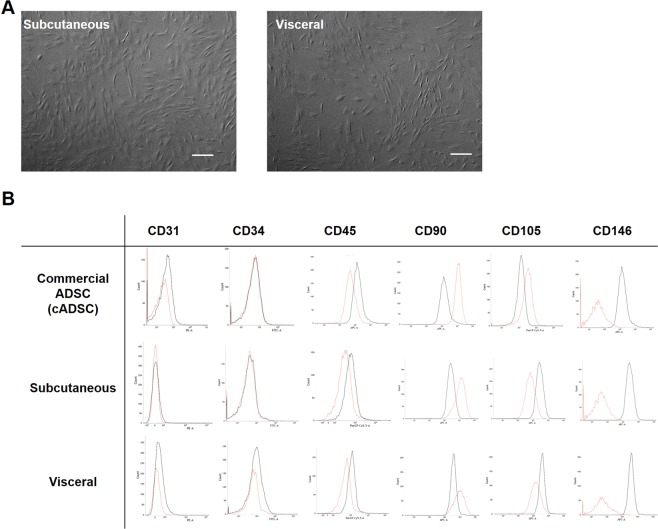
The characteristic differences of isolated ADSCs. (**A**) There were no differences for isolated ADSCs in morphology. Scale bar; 300 µm. (**B**) In FACS analysis, the expressions of the ADSCs were CDCD31^−^CD34^−^CD45^−^CD90^+^CD105^−^CD146^−^ in both the subcutaneous and visceral ADSCs. The expressions of the commercially provided ADSCs were CD31^−^CD34^−^CD45^−^CD90^+^CD105^+^CD146^−^, showing a difference between the two in CD105 only. Red line: antibody, black line: isotype.

For the isolated ADSCs there were no differences in morphology between those from the subcutaneous and the visceral adipose tissues (Fig. 1A). On the one hand, in the FACS analysis, the results of CD31^−^CD34^−^CD45^−^CD90^+^CD105^−^CD146^−^ were the same in both the subcutaneous and visceral ADSCs. On the other hand, the results were CD31^−^CD34^−^CD45^−^CD90^+^CD105^+^CD146^−^ for the commercially available ADSCs, showing a difference between the two in only the CD105 component (Fig. 1B).

*The cytokine release pattern was different between the ADSCs from the subcutaneous and visceral adipose tissues*.

**Figure 2:**
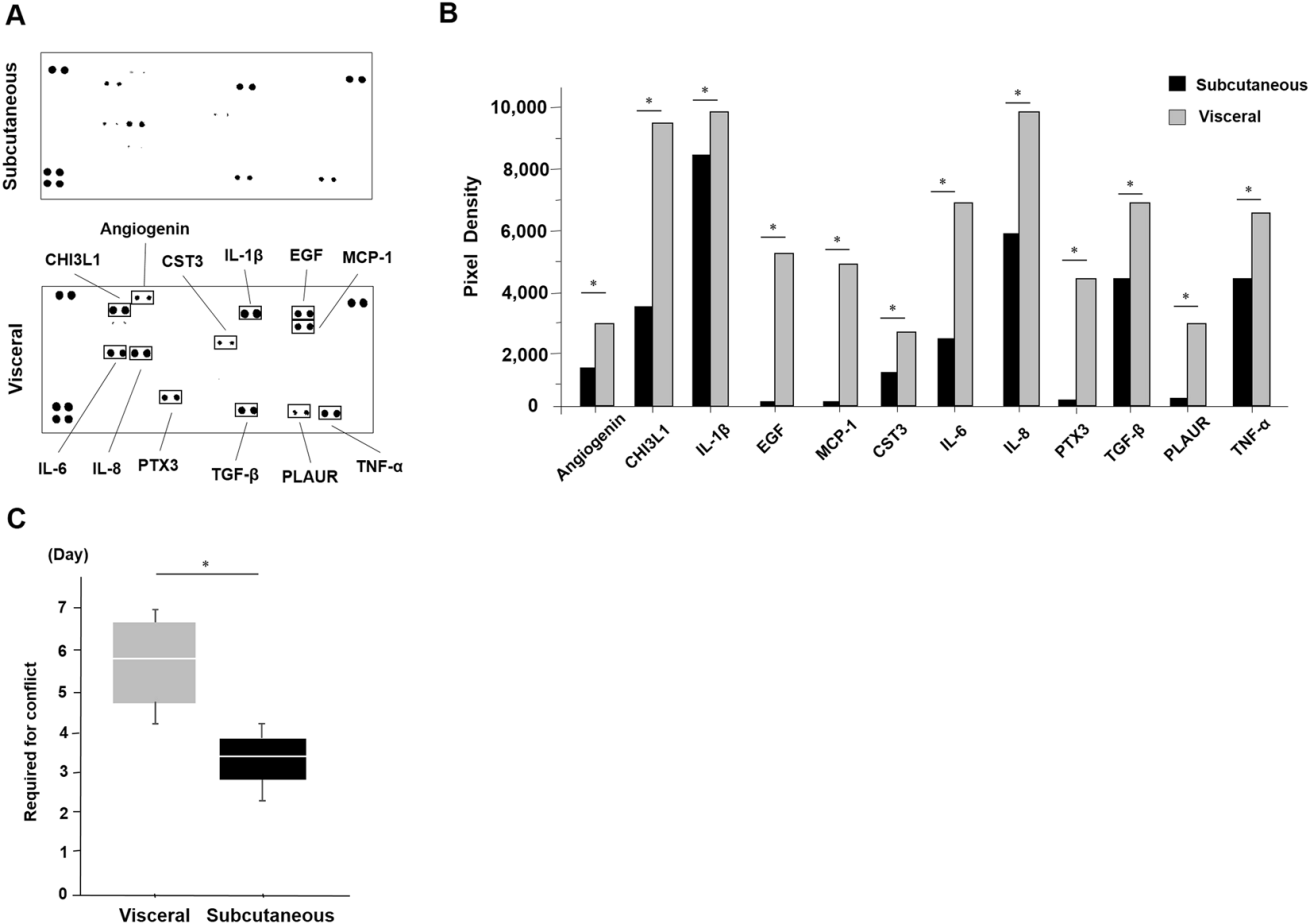
The differences in the cytokine release patterns between the ADSCs from the subcutaneous and visceral adipose tissues. (**A**) There were differences in some cytokines between the subcutaneous and visceral ADSCs using a cytokine assay kit. (**B**) These cytokines were angiogenesis-related, such as: CHI3LI; IL-1β; EGF; MCP-1; CST3; IL-6; IL-8; PTX3; TGF-β; PLAUR; and TNF-α. These cytokines’ secretions were smaller in the supernatants of the ADSC conditioned medium from subcutaneous adipose tissue. We analysed pixel density in each spot of the array (*P < 0.05, Mann-Whitney U test). **(C)** The cell proliferation speed was faster in the subcutaneous ADSCs than in the visceral ADSCs (3.3 vs. 5.8 days, *P = 0.04. Mann-Whitney U test).

Next, the cytokine release of each of the ADSCs was examined using the supernatants of the culture conditioned medium (Fig. 2A). There were differences in some cytokines between the subcutaneous and visceral ADSCs such as angiogenesis and inflammatory cytokines. These cytokines were: Angiogenesis; Chitinase 3-like 1 (CHI3LI); Interleukin-1β (IL-1β); Epidermal growth factor (EGF); Monocyte chemoattractant protein-1 (MCP-1); Cystatin C (CST3); Interleukin-6 (IL-6); Interleukin-8 (IL-8); Pentraxin 3 (PTX3); Transforming growth factor-β (TGF-β); Plasminogen activator urokinase receptor (PLAUR); and Tumour necrosis factor-α (TNF-α) (Fig. 2B). These cytokines’ secretions were decreased in the supernatants of the ADSCs conditioned medium from the subcutaneous adipose tissue compared to that from the visceral adipose tissue (p < 0.05, Mann-Whitney *U* test, Fig. 2B). Moreover, we measured the days until 10 cm dish is confluent as the proliferation ability. It was higher in the subcutaneous ADSCs than that of the visceral ADSCs (3.3 days vs. 5.8 days, p = 0.04, Mann-Whitney *U* test, Fig. 2C). In terms of the growth rate, the subcutaneous ADSCs proliferated 1.75 times faster than the visceral ADSCs.

### The cell qualities of the generated IPCs

**Figure 3:**
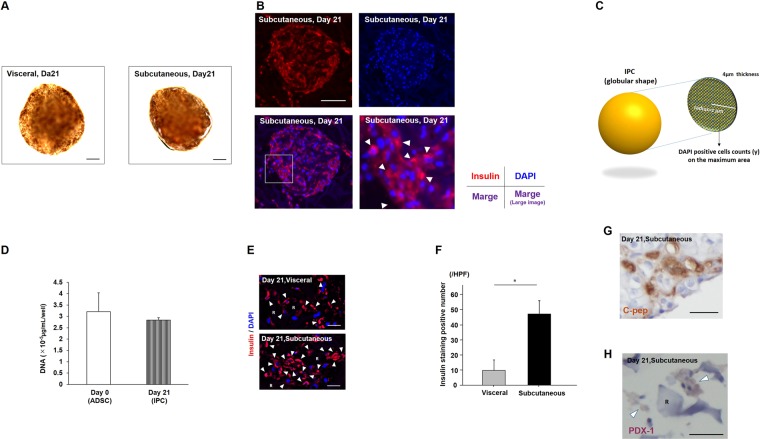
The characteristic differences of induced IPCs. (**A**) There were no differences for induced IPCs in morphology. Dithizone staining. Scale bar; 30 µm. (**B**) Insulin positive cells were confirmed using immunofluorescence staining. Immunofluorescence staining: Insulin (red, upper left); nucleus (blue, DAPI, upper right), merged image (lower left). R: RCP γ piece. The white square corresponded to a larger image (lower right). White arrowheads showed positive staining of insulin in the cytoplasm of cells. Scale bar; 50 µm. (**C**) The schema of methods for the calculation of cell numbers in IPCs. DAPI positive cells on 4 µm thickness were counted for using immunofluorescence images. Assuming that an IPC was a globular shape, that cell density was homogeneous and that the cutting plane was a perfect circle with the radius of z µm, the estimated IPC cell numbers can be found using this formula. A cell count on the cutting plane estimated the IPC cell numbers = z^2^π × 4: 4/3π × (average IPC diameter)^3^. (**D**) DNA quantity of cells on day zero (0) (ADSC) and day twenty-one (21) (IPC). Three independents experiments. (**E**) Insulin immunofluorescence (red: insulin, blue: DAPI, R: RCP γ piece, white arrowheads: positive staining of insulin in cytoplasm of cells). Scale bar; 5 µm. (**F**) Insulin staining positive cell numbers in random three high power fields were increased in the subcutaneous IPCs (43.0 vs. 5.0/high power field, *P = 0.01, Mann-Whitney U test). Cytoplasm of many cells in IPCs were strongly stained by C-peptide (**G**) and PDX-1(G). R: RCP γ piece, white arrowheads: positive staining of cells, Scale bar; 10 µm.

There were no differences for the induced IPCs in morphology between those from the subcutaneous and the visceral adipose tissues (Fig. 3A). In insulin and 4′, 6-diamidino-2-phenylindole (DAPI) immunofluorescence, the cytoplasm of the generated IPCs was well stained on day twenty-one (21) (Fig. 3B). To estimate the number of generated IPCs the DAPI positive cells when counted showed an average count (n = 3) of 163. Thus it was calculated that there were 1.6 × 10^4^ cells in one IPC when an IPC was assumed to have a 300 μm diameter globular shape (Fig. 3C). As well, the number of cells was estimated by the quantity of the DNA of the ADSCs which was 88.4% preserved in the generated IPCs (from 3.2 × 10^6^ μg/mL/plate to 2.8 × 10^6^ μg/mL/plate, the average of three (3) independent plates, Fig. 3D). Thus it was calculated that, theoretically, 1.8 × 10^4^ cells existed in one IPC. Comparing generated IPCs from fat tissue procurement cites, there was a difference between the numbers of strongly insulin positive cells in the IPCs derived from the subcutaneous and from the visceral ADSCs (43.0 vs. 5.0/high power field, *P* = 0.01, Mann-Whitney U test, Fig. 3E,F). In these generated IPCs the cytoplasm of many of the cells was well stained by C-peptide and PDX-1 antibodies on day twenty-one (21) (Fig. 3G,H).

### The difference of generated IPCs for glucose stimulation

**Figure 4:**
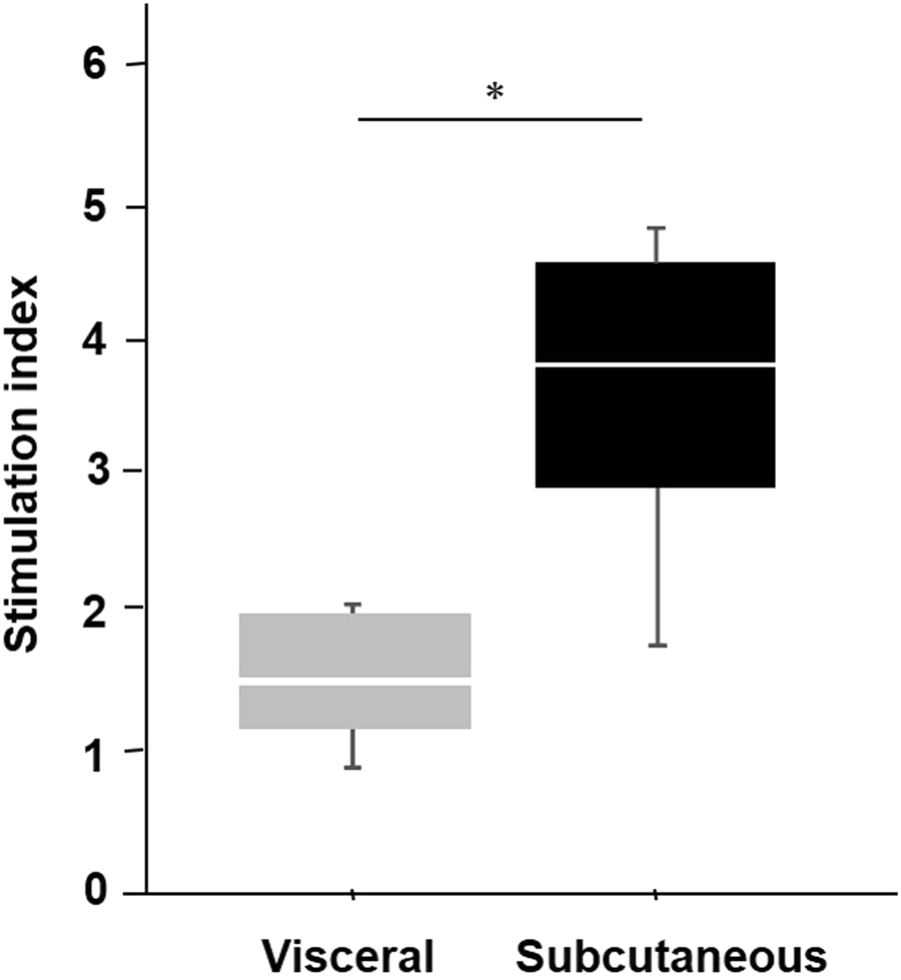
GSIS test of IPCs. The insulin secretion capacity as a glucose stimulation test was higher in the subcutaneous IPCs than the visceral IPCs (3.8 vs. 1.5, *P = 0.02, Mann-Whitney U test).

Next, the differences between the IPCs generated from the ADSCs isolated from the subcutaneous and the visceral adipose tissues were examined. A glucose stimulation test of the IPCs at day twenty-one (21) showed the insulin secretion capacity was higher in the subcutaneous IPCs than the visceral IPCs (subcutaneous IPC: 4.81, 3.84, 4.67, 1.86; visceral IPCs: 0.90, 1.86, 1.34, 2.02; median SI values of subcutaneous IPC vs visceral 3.8 vs. 1.5; *P* = 0.02, Mann-Whitney *U* test, Fig. 4).

### *In vivo* functional assessment of the IPCs

**Figure 5:**
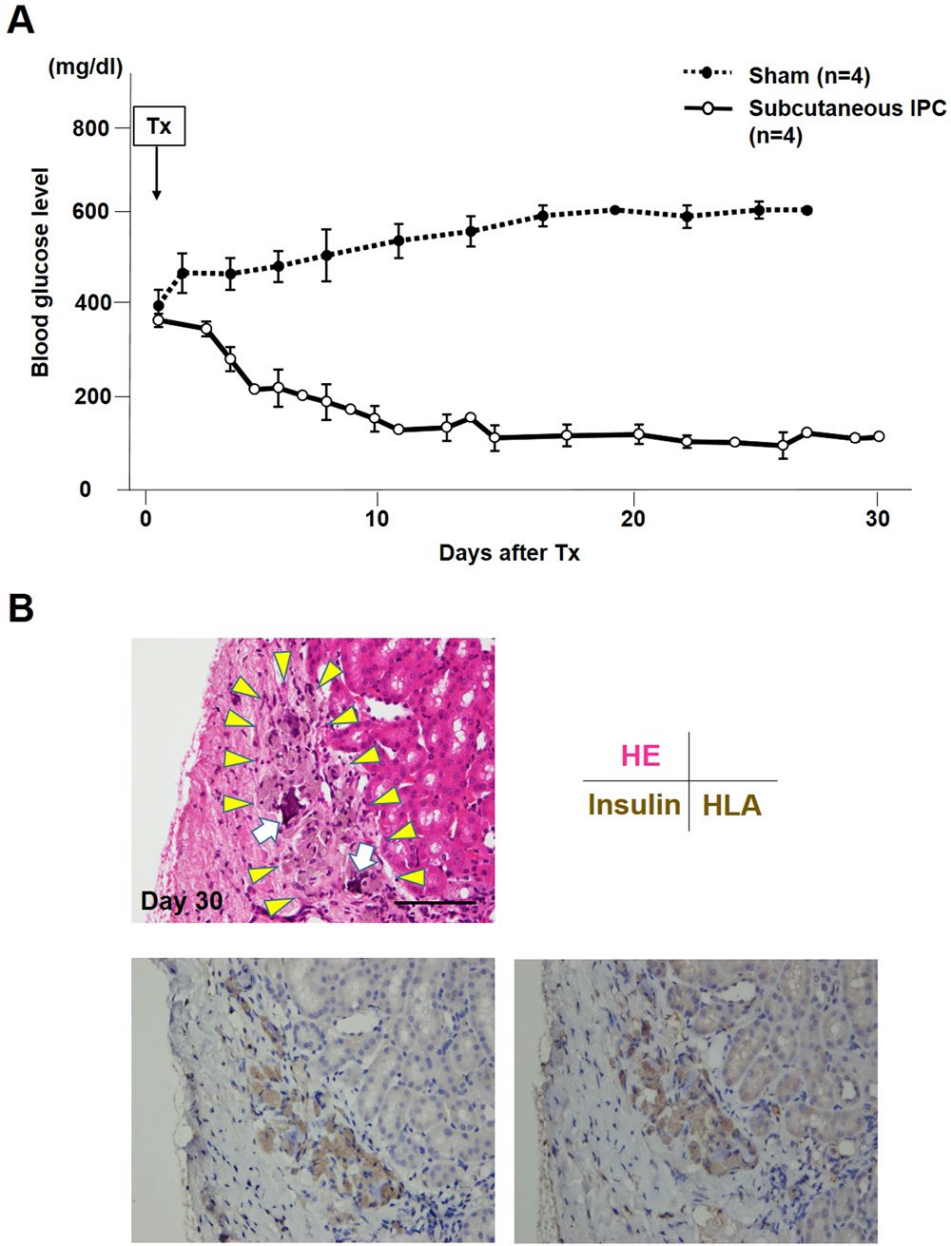
*In vivo* functional test. (**A**) Ninety-six (96) IPCs generated from the ADSCs and isolated from the subcutaneous adipose tissue were transplanted under the kidney capsule of streptozotocin-induced diabetic nude mice (n = 4, each mouse received IPCs from a different patient). In the sham group (n = 4), the blood glucose level remained at a high level. However, in the IPC-Tx group, the non-fasting blood glucose level decreased gradually after transplantation and remained normal. (**B**) Transplanted IPCs were observed under the kidney capsule on day thirty (30) post-transplantation (HE stains, upper left, yellow arrowheads). RCP pieces were still observed in these clusters (HE stains, upper left, white arrows). These clusters were well stained by anti-insulin antibody (lower left) and anti-human leukocyte antigen (HLA) class I (lower right). Scale bar; 30 µm.

From the results above, the *in vivo* function of IPCs derived from the ADSCs isolated from subcutaneous fat tissue was investigated. The non-fasting blood glucose levels of the recipients (n = 4) are shown in Fig. 5A. In the sham group (n = 4), the blood glucose levels only increased and were never marked below 400 mg/dl throughout the experiment. In contrast, the blood glucose levels decreased gradually in the IPC group to below 200 mg/dl by day nine (9) after transplantation and stayed around 100 mg/dl up to thirty (30) days after transplantation (4/4, 100%).

### Pathology of the transplanted IPCs

The transplanted IPCs were observed under the kidney capsule on day thirty (30) post-transplantation. RCP pieces were still observed in these clusters (Fig. 5B, upper). These clusters were well stained by anti-insulin and anti-human leukocyte antigen (HLA) class I (Fig. 5B, lower).

## Discussion

ITx is potentially a curative treatment for people with TIDM. However, it currently requires multi-donors to secure enough islets to transplant into one recipient to achieve a successful outcome. Thus, the generation of IPCs has the possibility to resolve these urgent issues and ADSCs can be obtained by a less invasive procurement method with lower ethical problems. As the location to harvest ADSCs, this study focused on subcutaneous adipose tissue because of the safety and simplicity of procurement. It can be collected by local anaesthesia and can be often used from the disposal waste of plastic surgery (as stromal vascular fraction). Also, it is reported ADSCs derived from subcutaneous adipose tissue can be regenerated and used by dermatology and in plastic surgery^17,18^. However, abdominal surgeons generally use omentum (visceral adipose tissue) for the repair of gastrointestinal perforations^19,20^. The authors have also experienced that these perforative points were completely replaced with regenerated tissues by an omental patch, thus digestive surgeons often have the impression that omentum has superior self-renewal ability. Moreover, it has also been reported that omental ADSCs promote angiogenesis and cell proliferation^21^. Therefore, a major question focused on in this study was whether isolated ADSCs from subcutaneous adipose tissue are better than visceral fat tissue for generating superior functioning IPCs. At first, the surface markers of the ADSCs isolated from subcutaneous and visceral fat tissue were the same, even though CD105 was negative compared to the commercially available ADSCs. It is reported that CD105 is a co-receptor for transforming the growth factor beta (TGFβ) receptor that antagonizes TGFβ signalling and is also a marker for vascular endothelial cells^22^. Moreover, it is also reported that freshly isolated ADSC contains various cell types, and homogeneous CD105^+^ cells only remain after many passages on plastic dishes^23^. However, CD105^+^ and CD105^−^ MSC subpopulations varied in their differentiation and immune-regulatory properties^24^. According to this report, CD105^−^ MSCs were more prone to differentiate and suppress the proliferation of CD4^+^ T cells compared to CD105^+^ MSCs. Moreover, the CD105^−^ cells showed superior differentiation potential in chondroid lesions compared to the CD105^+^ cells^25^. Thus, the finding in this study may suggest that CD105^−^ADSCs could affect the differentiation potential toward IPCs, even though investigations should continue regarding the fate and cytokine secretory ability of CD105^−^ADSCs.

Regarding to cytokines, subcutaneous ADSCs were found to secret less fat-tissue related inflammatory cytokines and had better proliferation abilities. On the one hand, the IPCs from subcutaneous ADSCs showed better insulin secretive function and expressed more insulin levels *in vitro*, and converted the hyperglycaemic state of STZ-induced diabetic nude mice into normo-glycaemia *in vivo*. Originally, IPCs should be induced for each harvest location, and performed *in-vivo* functional test to compare the function. However, IPCs derived from the visceral adipose tissue still had a low SI level on day 21 and we considered that IPCs (Day 21) derived from them are not suitable for transplantation experiments because of immature condition (we previously examined that IPCs less than SI 3 is not resulted in normalizing blood glucose after transplantation, data not shown). So, we showed only transplantation results of IPCs derived from Sham and subcutaneous adipose tissue.

On the other hand, the visceral ADSCs were exposed to more inflammatory cytokines and the IPCs generated from them showed poor insulin secretion. Regarding characteristic differences, subcutaneous adipocytes secrete adiponectin and leptin which are regarded as low-density cholesterol, while visceral adipocytes secrete inflammatory cytokines such as: IL-6; TNF-α; MCP-1; PAI-1; and angiotensin II^26^. It was reported that transforming growth factor-β (TGF-β) secreted from MSCs caused a decrease in cell viability and differentiation potential^27,28^. In addition, multiple signalling cascades stimulated by interleukin-1β (IL-1β), interferon-γ (IFN-γ), and tumour necrosis factor-α (TNF-α) incited inducible nitric oxide synthase (iNOS) and resulted in apoptosis and inhibited functionality^29,30^. This defect in cell activity is typically characteristic of impaired insulin biosynthesis and secretion, usually accompanied by oxidative and endoplasmic reticulum (ER) stress^31–34^. In summary, the ADSCs derived from visceral adipose tissue secret more inflammatory cytokines and possible exposure to more oxidative and ER stress. The IPCs generated from them showed poor differentiation and insulin secretion.

Moreover, when considering the proper transplantation site, it is proposed that the intra-mesentery transplantation identified in a previous study is one reasonable and less-invasive transplantation site^12^. As professional hepato-biliary-pancreatic and transplant surgeons, the authors routinely undergo high-risk laparoscopic surgeries^35–37^. Thus it may be safe and easy for both recipients and surgeons to introduce intra-mesentery IPC transplantation as a Phase I/II study after considering the number of cells administered because the injection of a small amount into the mesentery is an easy technique when commercially available laparoscopic instruments are used.

Taken together, this study showed the possibility of an innovation to create IPCs from subcutaneous adipose tissue which was proved to have a more rapid differentiation rate and better functional efficiency compared to visceral adipose tissue. Moreover, that might be completely in agreement with the authors’ future research. That is: the intention to obtain 1.0 cm^3^ of adipose tissue from T1DM patients; to isolate and passage ADSCs from this tissue (approximately 1.0 × 10^7^ cells); to differentiate the cells into IPCs (approximately 88% cell numbers maintained) using the modified protocol identified in this study; and culture them without any genetic modifications. The generated IPCs would then be auto-transplanted into the patients’ mesentery laparoscopically. Within such concepts, these results may concretize a strategy for the clinical application of IPCs.

## Materials and Methods

### Research settings

Four (4) patients who had undergone digestive surgeries at The Tokushima University from 2018 to 2019 were included in this study. Both subcutaneous and visceral adipose tissues were collected and disposed as not necessary during the surgeries. This study was authorized in advance by the Institutional Review Board of the Tokushima University Hospital (the approved ID number: 3090) and the University Hospital Medical Information Network (the approved ID number: 000035546), was performed in accordance with relevant guidelines/regulations. All patients provided written informed consents.

### Cell preparation

0.1 gram of adipose tissue was harvested from each patient’s subcutaneous tissue and hepatic round ligament in the sterile environment during operation. ADSCs were obtained two (2) weeks later using a adipose stem cell isolation kit (Funakoshi Co, Tokyo, Japan) and passaged with ADSCs basal medium mixture of MesenPRO^TM^ RS (Gibco, Carlsbad, CA) and GlutaMAX^TM^-I (Gibco). After three (3) passages, the ADSCs were counted and mixed with recombinant peptide micro-pieces (RCP μ-pieces, FUJIFILM, Tokyo, Japan), and injected into all of the wells in a Nunchlon Sphera 96U Bottom Plate (Thermo fisher scientific, Waltham, MA) as a 3D culture. The protocol for induction of IPCs was modified from the authors’ previous reported protocol^9^. Specifically, the contents of the differentiation cocktail were changed to recombinant human activin-A, hepatocyte growth factor and human albumin. The ADSCs were cultured using the Step-1 (from day 0 to day 7) medium containing: Dulbecco’s modified Eagle’s medium/F12 (Gibco); 1% recombinant human albumin (Wako, Osaka, Japan); exendin-4 (Sigma-Aldrich, St. Louis, MO); 1% N2 supplements (Gibco); 1% B27 supplements (Gibco); and 50 ng/mL recombinant human activin-A (PeproTech Inc., Rocky Hill, NJ). Step-2 (from day 8 to day 21) was prepared the same way as Step-1 medium with the addition of: 50 ng/mL recombinant human hepatocyte growth factor (PeproTech Inc.); valproic acid (Wako); and 10 mM nicotinamide (Sigma-Aldrich). The culture medium was changed and supernatant was collected every two (2) days. STEMPROTM Human Adipose-Derived Stem Cells (Invitrogen, Grand Island, NY) was purchased and used for fluorescence activated cell sorting (FACS) analysis.

### FACS analysis

2.0 × 10^6^ ADSCs were washed twice with Phosphate Buffered Saline (PBS) (SANTA CRUZ Bio., Dallas, TX) and added to 100 μL of FACS buffer and 5 μL of each antibody: CD31 (Thermo fisher scientific); CD34 (Thermo fisher scientific); CD45 (BioLegend, San Diego, CA); CD90 (Thermo fisher scientific); CD105 (Thermo fisher scientific); and CD146 (BioLegend). After thirty (30) minutes in a dark room, the cells were analysed using FACSVerse and BD FACSuite software (BD Biosciences, San Paulo, Brazil).

### Glucose-stimulated insulin secretion (GSIS) test

To determine the *in vitro* potency of the IPCs, the insulin secretory response to glucose was measured using a modified method previously described^38–40^. Briefly, 10 IPCs were randomly transferred to a cell culture insert with: Krebs buffer (115 mM NaCl; 5.0 mM KCl; 2.3 mM CaCl_2_; 1.0 mM MgCl; 1.2 mM KH_2_PO_4_; 25 mM NaHCO_3_; pH 7.4); 25 mM HEPES (Gibco); and 0.1% BSA fraction V (Sigma-Aldrich) containing 2.8 mM glucose and incubated at 37 °C for one (1) hour as pre-incubation. Thereafter, the IPCs were suspended three (3) times for one (1) hour at 37 °C in Krebs buffer with the addition of various glucose concentrations (basal I: 2.8 mM, stimulation: 22 mM, respectively). The insulin level was measured using an Insulin Enzyme-Linked Immunosorbent Assay Kit (AKRIN-011T, Wako Shibayagi Corporation, Gunma, Japan).

### Immunofluorescence and confocal microscopy

The IPCs in the culture medium were fixed with iP gel^®^ (Nippon Genetics Corporation, Tokyo, Japan) and 10% formalin (Gibco), embedded in optical cutting temperature compound and frozen. Then 4-µm-thick sections were cut, rinsed in PBS and 5% BSA, and incubated with Universal Blocker Reagent for thirty (30) minutes in a humidified chamber at room temperature. Thereafter, sections were incubated with primary antibodies, anti-insulin (1:100 dilution; 4590, Cell Signaling Technology, Tokyo, Japan) overnight at 4 °C in a humidified chamber. After being washed with PBS and 4′, 6-diamidino-2-phenylindole (DAPI; Invitrogen Corp, Carlsbad, CA) the sections were applied to the slides and incubated at room temperature for two (2) hours in the dark. The cellular composition was determined by manually counting the stained cells using a fluorescent microscope (Keyence, Keyence Corp, Chicago, IL).

### Cell number estimation of IPCs by DNA quantity

DNA quantity was measured using a DNA Quantity kit (Cosmo Bio, Tokyo, Japan) according to the manufacturer’s protocol. Briefly, ADSCs and IPCs were washed with phosphate-buffered saline (PBS) and homogenized ultrasonically after a 0.5 ml buffer was added to each well. Then absorbance at 450 nm was measured using a plate reader (SpectraMax i3; Molecular Devices, Tokyo, Japan) at a correction wavelength of 540 nm.

### Cell count of IPCs (mathematically)

DAPI positive cells on 4 µm thickness were counted for using immunofluorescence images. Assuming that an IPC was a globular shape, that cell density was homogeneous and that the cutting plane was a perfect circle with the radius of z µm, the estimated IPC cell numbers can be found using this formula. A cell count on the cutting plane: estimated the IPC cell numbers = z^2^π × 4: 4/3π × (average IPC diameter)^3^.

### Dithizone staining

The cultured cells were stained using a dithizone solution. The dithizone solution consisted of 50 mg dithizone (Wako) per 5 mL dimethyl sulfoxide (Wako). The IPCs were incubated in dithizone solution under 37 °C and 5% CO_2_ conditions after being washed by PBS three (3) times. Stained samples were investigated using a multi-purpose microscope BZ-X710 (KEYENCE Engineering, Japan) and BZ-X analyser (KEYENCE Software, Japan).

### Cytokine assay

A Human XL Cytokine Array Kit (R&D SYSTEMS., Minneapolis, MN) was used to measure each cytokine secretion of the ADSC’s conditioned medium. The ADSCs were confluented and harvested in each conditioned medium. The procedure used was in accordance with the cytokine assay protocol. Briefly, each membrane was placed in a separate well and incubated with the array buffer for one (1) hour. Samples were prepared by diluting the desired quantity with the array buffer. After aspirating the array buffer and adding prepared samples, they were incubated overnight at 2–8 °C on a rocking platform shaker. Each membrane was washed and added to the diluted detection antibody cocktail then incubated for one (1) hour. After incubation, the prepared Chemi Reagent Mix was inserted onto each membrane. The membrane was exposed to X-ray film and analysed pixel density in each spot of the array.

### IPCs transplantation

Eight (8) week-old male BALB/c nu-nu mice (Charles River Laboratories, Kanagawa, Japan) were used as the recipients (n = 4). The mice were allowed free access to water and standard laboratory food and were housed at a temperature of 22 ± 2 °C, relative humidity of 55 ± 5%, and a twelve (12) hour light: twelve (12) hour dark cycle with lights. The present study was conducted in compliance with the Division for Animal Research Resources, Graduate School of Biomedical Sciences, Tokushima University (approved number: T29-29). The experiments and procedures were approved by the Animal Care and Use Committee of the University of Tokushima and were performed in accordance with the NIH Guide for the Care and Use of Laboratory Animals.

To induce a diabetic state in the mice, 200 mg/kg streptozotocin (STZ, Sigma-Aldrich) was administered intra-peritoneal as a single injection. The diabetic states were defined after two (2) consecutive measurements of blood glucose over 350 mg/dl or one (1) measurement over 400 mg/dl with a glucometer (Terumo Corporation, Tokyo, Japan). Then, ninety-six (96) IPCs were carefully hand-picked and transplanted under the kidney capsule of the streptozotocin-induced diabetic nude mice (n = 4, each mouse received IPCs from a different patient). The non-fasted glucose levels of all the mice were measured from the tail vein every one (1) or two (2) days after surgery.

### Immunohistochemical staining

Generated cells and extirpated organs were prepared and stained by methods already reported^12^. Briefly, generated IPCs were fixed using a cell fixing kit (Funakoshi), recipient mice were sacrificed and IPCs bearing kidneys were extirpated. 4 μm thick paraffin embedded sections were incubated with primary antibodies against insulin (aa287–299, LS-B129; LSBio) at a dilution of 1:100 in phosphate-buffered saline (PBS), anti-HLA class I (ab70328; abcam) 1:100 in PBS, C-peptide (bs-0274R, Funakoshi) 1:100 in PBS and pancreatic and duodenal homeobox 1 (PDX-1, ab47308;abcam) 1:200 in PBS for 1 h at room temperature after deparaffinization and antigen retrieval. Slides were then incubated with biotinylated secondary antibody, followed by treatment with a streptavidin-biotin-horseradish peroxidase complex. Positive staining was visualized with diaminobenzidine and cell nuclei were counterstained with Mayer’s haematoxylin.

### Statistical analysis

The data analysis was performed with statistical software (JMP software, version 13; SAS Campus Drive, Cary, NC). Comparisons between the two (2) groups were performed by Mann-Whitney *U* test. The One-way ANOVA with Turkey-Kramer’s test was used to compare *in vivo* functional test. In the figures, the median values (75th and 25th percentiles) and median ± standard deviation (SD) are given, respectively. A value of *p* < 0.05 was considered to indicate statistical significance.

## Data Availability

The datasets used and/or analyzed in this study are available from the corresponding author upon reasonable request.
